# Small Bowel Enteritis and Myocardial Depression Complicating Organophosphorus Poison in a Patient With Sleeve Gastrectomy and Intestinal Bypass Surgeries: An Atypical Presentation

**DOI:** 10.7759/cureus.35522

**Published:** 2023-02-27

**Authors:** Mohammed Shaban, Franklin Sosa, Muhammad Yasir Anwar, Irhoboudu Dickson Atogwe, Misbahuddin Khaja

**Affiliations:** 1 Internal Medicine, BronxCare Hospital Center, Icahn School of Medicine at Mount Sinai, New York, USA; 2 Medicine, BronxCare Hospital Center, Icahn School of Medicine at Mount Sinai, New York, USA; 3 Internal Medicine, King Edward Medical University, Lahore, PAK; 4 Internal Medicine/Pulmonary Critical Care, BronxCare Hospital Center, Icahn School of Medicine at Mount Sinai, New York, USA

**Keywords:** intestinal bypass, enteritis, sleeve gastrectomy, myocarditis, organophosphorus

## Abstract

Organophosphate (OP) is a pesticide that has been used in agriculture and domestic pest control since the mid-1900s. Acute OP toxicity is caused by inhibiting the acetylcholinesterase (AChE) enzyme, resulting in a cholinergic surge. It is treated with atropine and pralidoxime. Our case is a patient with a past history of sleeve gastrectomy and intestinal bypass surgery presented after oral OP intake. He initially had small bowel enteritis, followed by lactic acidosis, acute renal injury, and distributive shock. The serum troponin had peaked 50-folds. The echocardiography showed myocardial depression and global hypokinesia with no significant wall motion abnormalities. In contrast to classic bradycardia with OP poisoning, our patient developed persistent sinus tachycardia on the second day. He had a concomitant alcohol withdrawal syndrome, which was managed with intravenous (IV) hydration and benzodiazepines. He had a dramatic improvement on the third day with near resolution of creatinine and lactic acid. The outpatient cardiac follow-up showed partial resolution of the left ventricular ejection fraction (EF) to 48%.

In this literature, we discuss the complications and long-term effects of bariatric surgeries, particularly on gastric emptying and medication absorption. We also discuss OP mechanism of action, clinical presentation, therapeutic lines, and atypical presentations in the prior literatures.

## Introduction

Dichlorvos, an organophosphorus (OP) compound, is a predominant pesticide used in domestic insect control. Acute and prolonged exposure may lead to neurological, carcinogenic, immunological, respiratory, metabolic, and dermal effects. Its toxicity is caused by inhibiting acetylcholinesterase (AChE) at the cholinergic junction of the nervous system [1]. Acute OP poisoning can cause cardiac complications such as QT interval prolongation, ST-T changes, U waves, ventricular premature contractions, and episodic bradycardia [2]. Acute OP poisoning is also reported to cause toxic myocardial depression by direct toxicity or cholinergic overstimulation syndrome [3].

Here, we present a case report of elemental intake of organic phosphorus in a patient with a history of gastric bypass surgery causing small bowel enteritis, myocardial depression, acute renal failure, and distributive shock.

## Case presentation

A 42-year-old male patient with a past medical history of hypertension, alcohol abuse disorder, smoking, sleeve gastrectomy in 2014, and gastric bypass in 2017 presented to the emergency room with diffuse abdominal pain a few hours after suicidal intake of dichlorvos (2,2-dichlorovinyl dimethyl phosphate or DDVP). He was vitally stable with blood pressure (BP) of 110/81 mmHg, heart rate (HR) of 96 beats per minute (bpm), and respiratory rate of 20/minute. His abdominal pain was associated with generalized tenderness; otherwise, his physical examination was unremarkable. The initial blood workup is shown in Table 1.

**Table 1:** The admission's laboratory workup INR: international normalized ratio

Test	Value (reference)	Test	Value (reference)
Sodium, serum	138 (135-145 mEq/L)	Hemoglobin	15.3 (12-16 g/dL)
Potassium, serum	4.6 (3.5-5 mEq/L)	Leukocytic count	10.6 (4.8-10.8 k/ul)
Chloride, serum	97 (98-108 mEq/L)	Platelet count	376 (150-400 k/ul)
Bicarbonate, serum	12 (24-30 mEq/L)	Total protein, serum	8.5 (5.8-8.3 g/dL)
Blood urea nitrogen	7 (6-20 mg/dL)	Albumin, serum	5.2 (3.2-4.6 g/dL)
Creatinine, serum	1.1 (0.5-1.5 mg/dL)	Aspartate transaminase, serum	27 (9-36 unit/L)
Magnesium, serum	2.5 (1.5-2.7 mg/dL)	Alanine aminotransferase, serum	18 (4-50 unit/L)
Osmolality, serum	341 (275-295 mosm/kg)	Total bilirubin, serum	0.4 (0.2-1.1 mg/dL)
Calcium, serum	8.9 (8.5-10.5 mg/dL)	Direct bilirubin, serum	<0.2 (0.0-0.3 mg/dL)
Ethanol level, serum	206 (≤10 mg/dL)	INR	1.05 (0.85-1.14)
Acetylsalicylic acid level, serum	<0.3 (3.0-10.0 mg/dL)	pH, blood gas	7.214 (7.350-7.450)
Acetaminophen level, serum	<5.0 (10.0-30.0 ug/mL)	pCO_2_, blood gas	31.0 (35.0-45.0 mmHg)
Lactic acid	5.0 (0.5-1.6 mmol/L)	pO_2_, blood gas	80.4 (83.0-108.0 mmHg)

**Figure 1:**
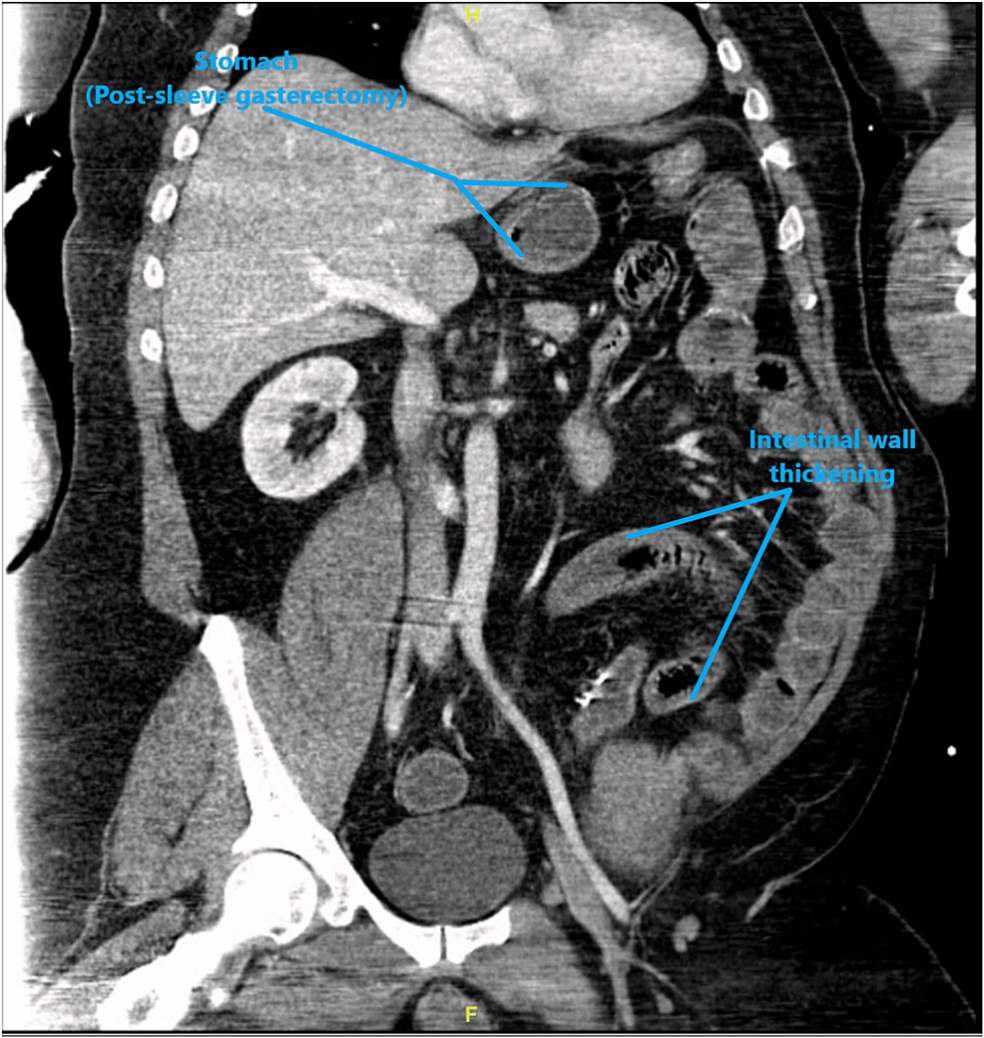
CT of the abdomen and pelvis with IV contrast with wall thickening of the small bowel (coronal section) IV: intravenous

**Figure 2:**
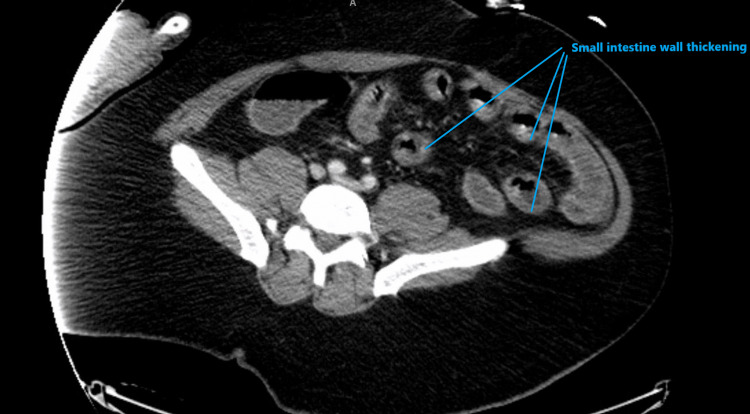
CT of the abdomen and pelvis with IV contrast showing the wall thickening of the small bowel IV: intravenous

The New York City Poison Control Center was contacted, and no atropine or pralidoxime was recommended, given the absence of bradycardia. A few hours later, the patient began to have profuse watery diarrhea; his blood pressure dropped to 82/59 mmHg, and his HR increased gradually to 170 bpm. The electrocardiogram (EKG) showed sinus tachycardia. The patient received fluid resuscitation with 2 L of isotonic crystalloids. The patient underwent CT of the abdomen and pelvis with intravenous (IV) contrast, which showed wall thickening of the small bowel, likely representing enteritis (Figures 1-2). Conservative management was started in terms of ceftriaxone and metronidazole.

Laboratory follow-up showed up-trending lactic acid dramatically to 6.6 mmol/L (Table 2). Additional 3 L of fluid resuscitation with close monitoring was continued in the intensive care unit (ICU). The serum creatinine peaked from 1.1 to 3.4 mg/dL in the first 24 hours before trending to baseline within 72 hours post admission. Lactic acid also returned to baseline values on the third day of admission. Urine chemistry pointed to prerenal etiology with urine osmolality 475 mosm/kg and urine sodium 35 mEq/L. The urine drug screen was negative for common illicit drugs in the community. The repeat CT with oral contrast showed wall thickening of the small bowel loops with subtle adjacent stranding and focal wall thickening of the splenic flexure. Fecal occult blood was positive with any evidence of active gastrointestinal (GI) bleeding.

**Table 2:** The trend of blood chemistry and blood gas during the initial three days

	pH	pCO_2_	HCO_3_	Sodium	Potassium	Chloride	Creatinine	Blood urea nitrogen	Lactic acid
Day 1	7.22	32	14	144	4.7	107	2.2	16	6.9
Day 2	7.27	38	13	143	3.8	110	3.4	28	2.3
Day 3	7.37	34	19	142	3.5	108	1.8	30	2

**Figure 3:**
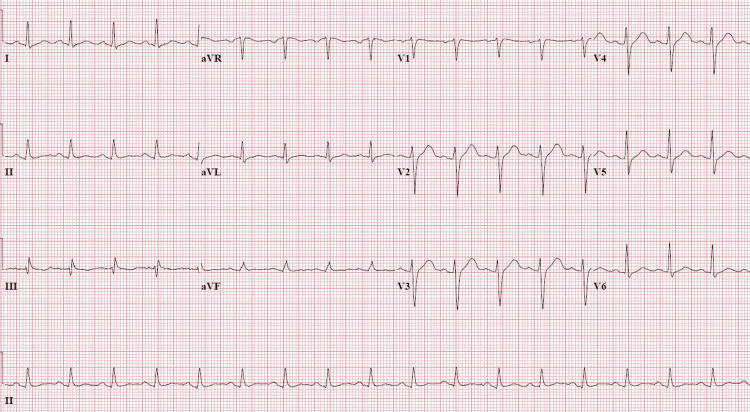
EKG shows non-specific ST-T wave changes EKG, electrocardiogram; aVR, augmented vector right; aVL, augmented vector left; aVF, augmented vector foot

The patient denied any cardiac symptoms over the last few years with non-specific ST-T wave changes in the initial EKG (Figure 3). The transthoracic echocardiogram (TTE) showed severe left ventricular systolic dysfunction with an ejection fraction (EF) of 36%. The perflutren contrast was used to enhance wall motion assessment and confirmed global hypokinesia without significant segmental variations. No significant valvular or right ventricular abnormalities were detected. The troponin T increased to 586 ng/L (standard reference is <12 ng/L) despite the absence of any EKG changes suggestive of an acute coronary syndrome. Inotropic support in the form of norepinephrine was required to maintain the blood pressure. The concomitant alcohol withdrawal syndrome was managed with benzodiazepines.

The hospital course was not remarkable for respiratory failure or clinical evidence of acute left ventricle (LV) decompensation. The patient's laboratory findings showed near-complete improvement on the fourth day of admission (Table 2). The cholinesterase activity was less than 50% confirming OP as an etiology of the current illness. All infectious workups came negative. Six weeks later, the follow-up in outpatient cardiology practice was relevant for the partial improvement of the EF to 48% and the absence of ischemia in the myocardial perfusion test.

## Discussion

Organophosphate (OP) is a pesticide used in agriculture and domestic pest control. The first OP insecticide was created in the mid-1800s but was widely used after World War II [4]. 2,2-Dichlorovinyl dimethyl phosphate (DDVP), also known as dichlorvos, is an organophosphate insecticide. Dichlorvos is the most commonly used organophosphate pesticide in developing countries. It is also used since the 1960s as an antihelminthic agent on dogs, horses, and swine and in fish farming to eradicate crustacean ectoparasites. Since 1992, the WHO has classified it as class 1B, a "highly hazardous" chemical [1]. OP causes its toxicity through the irreversible inactivation of acetylcholinesterase (AChE), the enzyme responsible for the breakdown of acetylcholine (ACh) in the plasma, red blood cells, and neuronal synapses in the peripheral and central nervous systems, causing the buildup of ACh, which in turn leads to the overstimulation of both the nicotinic receptors and the muscarinic receptors [5].

OP has widespread clinical varieties, including increased saliva and tear production, diarrhea, nausea, vomiting, small pupils, sweating, muscle tremors, and confusion. The onset of symptoms is often within minutes, and it can take weeks to disappear [4]. OP poisoning causes respiratory failure through central apnea and pulmonary dysfunction [6]. The vagal nerve tone has a crucial function in the central control of the respiratory rhythm through feedback signals from the lung to the brainstem, in addition to the peripheral regulation of pulmonary vasculature, airways, and secretions to maintain the pulmonary gas exchange [6]. As a result, the initial treatment for OP is atropine, an antimuscarinic agent, until the resolution of bronchospasm and respiratory secretions [4]. However, atropine only works on muscarinic-cholinergic receptors without affecting the nicotinic cholinergic receptors. The nicotinic receptors could be neutralized only by pralidoxime, which binds OP and reactivates the phosphorylated AChE [7]. Despite these therapies, the mortality rate can reach up to 25%, mainly because of respiratory failure [4].

Bariatric surgeries have been increasingly proving their value in managing obesity in adolescents and adults compared to traditional lifestyle and medical management. Gastric bypass and sleeve gastrectomy are the commonly used procedures for this purpose [8]. Approximately 60%-70% of obese patients are dyslipidemic, with a subsequent increased risk of developing cardiovascular diseases [9]. Studies demonstrated that these procedures are generally well tolerated, with an average body mass index loss between 25% and 29% [8]. Bariatric surgeries have proven their role in robust weight loss and reduced serum lipid levels. Gastric bypass surgery has shown remission of hyperlipidemia [9].

The perioperative mortality of bariatric procedures is less than 1%. The most common postsurgical complication is peritonitis from fistula formation in 1%-6% after gastric bypass and 3%-7% after sleeve gastrectomy [10]. Less frequently, particularly in a population with a hypercoagulable profile (such as antithrombin III, protein C and protein S deficiencies, or activated protein C resistance), acute post-procedural abdominal pain could be related to superior mesenteric vein thrombosis; appropriate early anticoagulation therapy is expected to improve the prognosis [10]. Other short-term (<30 days) complications include leaks, bleeding, and surgical site infections. The long-term complications are nutritional deficiencies [8]. Malnutrition is uncommon after restrictive surgery (ring, sleeve gastrectomy); however, it frequently occurs after malabsorptive surgery (bypass, biliary pancreatic shunt) due to changes in absorptive surface mechanisms [10].

Gastric sleeve procedures theoretically cause transit time and gastric acid secretion reduction, with resultant increased pH. In contrast, bypass procedures cause decreased absorption in the small intestine, reduced exposure to bile acids and enterohepatic circulation, and decreased gastrointestinal transit time [11]. Therefore, the pharmacokinetic prosperities of any ingested substance may be liable to absorption, distribution, metabolism, and elimination changes. The time to maximum concentration is often earlier, and this concentration may be higher with less consistent effects on trough concentrations and exposure [11]. However, contrary to these theories, Afshar et al. performed a study to assess bowel habits in patients after six months of gastric bypass or sleeve gastrectomy. They found a significant reduction in dietary fibers and bowel motion frequency. They believed decreased bowel motion frequency would prolong intestinal transit time [12]. Our patient's prior history of both gastric sleeve and bypass could be related to the odd pharmacokinetics, possibly dynamics of the ingested toxin. The atypical presentation of acute OP intoxication, such as tachycardia, enteritis, and myocardial depression, could be blamed on the disruptive anatomy of the GI tract. It was reflected in the management declining both atropine and pralidoxime; however, the outcome was the patient's survival with only supportive measures and benzodiazepines.

Acute OP poisoning can cause cardiac complications such as QT interval prolongation, ST-T changes, U waves, ventricular premature contractions, and episodic bradycardia. Patchy myocardial involvement was proved due to direct cardiac toxicity and patchy pericarditis, auricular thrombus, right ventricular hypertrophy, or dilatation [2]. Even though acute coronary syndrome is a rare finding of OP poisoning, it was reported in a 22-year-old male after ingesting monocrotophos poison. He presented with acute ST-elevation myocardial infarction; however, his coronaries had no gross coronary lesions [13]. Historically, to understand OP's direct cardiac toxicity, Dierkes-Tizek et al. tested the cardiac ATPase activity of rats after OP exposure. They found that OP had an inhibitory effect on the activities of the transport ATPase of the myofibers and mitochondrial ATPases. The Ca2+-stimulated ATPase was more sensitive to toxicity rather than the Na+/K+-ATPase. This inhibitory effect depended on the exposure duration, the dose of poison, and the OP agent used [14].

Benzodiazepines are generally recommended in acute OP poisoning as they antagonize some central signs and symptoms of cholinergic attack medicated by nicotinic receptors (i.e., insensitive to atropine) such as fasciculations, muscular spasms, seizures, anxiety, psychomotor agitation. In addition, benzodiazepines attenuate neuronal, cardiac, and muscular damage caused by cholinergic overstimulation [3]. Our patient received an adequate dose of benzodiazepine to manage concomitant acute alcohol withdrawal syndrome. However, he was not aware of any cardiac symptoms. The administered benzodiazepine may have masked the potentially acute cardiac pathology in the setting of acute OP poisoning.

Hypotension is a common complication of OP poison and is associated with increased in-hospital mortality, especially if advanced age, diabetic patient, or related to significant laboratory derangement [15]. The direct cardiotoxic effects of OP can partially explain hypotension, but the distributive shock still plays a role. The distributive shock was also reported in a young male after dichlorvos ingestion, where no other etiology of distributive shock could be identified, such as sepsis, anaphylaxis, burns, pancreatitis, toxic shock syndrome, spinal cord injury, or endocrine disorders. It was successfully managed with intravenous crystalloids, pressors, and atropine; however, delayed neuropathy remained evident after four weeks [16].

Peripheral vascular resistance is maintained at the arterioles, which dilates and constricts in response to different neuronal and hormonal signals. Generally, neurogenic/distributive shock occurs if there is a loss of sympathetic tone with resultant decreased cardiac output and a decrease in vascular resistance secondary to the loss of the arteriolar tone [17]. It might be possible that distributive shock here was secondary to acute neuritis supplying the vascular bed, which is responsible for maintaining the vessel tone and peripheral vascular resistance. Similarly, fluid resuscitation and short-term norepinephrine support dramatically improved our patient's distributive shock, lactic acidosis, and renal failure. Interestingly, our patient was tachycardic shortly after he was admitted to the ICU, contrary to the bradycardia expected in OP poisoning, pointing to direct myocardial effect and autonomic dysfunction. Autonomic dysfunction is reported with chronic low-level OP exposure; however, it is unclear after acute OP intoxication [18]. To study autonomic function after an acute OP overdose, Jayasinghe and Pathirana (2012) evaluated the autonomic function in 66 patients one and six weeks after acute exposure to OP. They studied the heart rate response to standing and the Valsalva maneuver, blood pressure variability, amplitude and latency of sympathetic skin response, pupil size, and post-void urine volume. They generally concluded the absence of long-term autonomic dysfunction following OP acute exposure [18].

Acute kidney injury as a direct sequela of OP poisoning was infrequently reported in the literature. The exact mechanism of renal damage is unclear [19,20]. We found two cases (a 17-year-old adolescent female [19] and a 58-year-old male [20]) in whom acute hemodialysis was temporarily needed until the recovery of renal function. In the second case [20], the inciting agent was accidental inhalation of OP; however, the course was more aggressive and complicated, with acute anuric renal failure and severe metabolic acidosis [20]. Our patient had an episode of acute renal failure a few hours after the IV contrast for the CT of the abdomen. Luckily, it resolved within 72 hours with aggressive fluid resuscitation. We do not point to the OP as inciting factor; however, the fact that OP can induce acute autonomic dysfunction with the alternation of vascular tone can aggravate the sharp rise of creatinine before the rapid return to baseline.

## Conclusions

OP has widespread clinical varieties with profuse secretion such as salivation and diarrhea, respiratory secretion, respiratory failure, and death. Bradycardia is the clinical finding directing atropine and oxime therapy to antagonize the cholinergic surge. In contrast to classic bradycardia with OP poisoning, our patient developed persistent sinus tachycardia, myocardial depression, and distributive shock. His ICU course was also complicated with small bowel enteritis, lactic acidosis, and acute renal failure. He had a dramatic improvement on the third day with intravenous hydration and benzodiazepines. The disruptive GI anatomy from the past sleeve gastrectomy and intestinal bypass surgeries possibly relates to the atypical OP presentation. The inciting pathology was confirmed to be acute OP by low AChE of less than 50%. Benzodiazepine with aggressive fluid resuscitation was the key to antagonizing the central effects of the cholinergic surge, maintaining intravascular volume, and reverting lactic acidosis in our clinical situation.
